# Effects of Coefficient of Thermal Expansion and Moisture Absorption on the Dimensional Accuracy of Carbon-Reinforced 3D Printed Parts

**DOI:** 10.3390/polym13213637

**Published:** 2021-10-21

**Authors:** Jessica L. Faust, Peter G. Kelly, Bruce D. Jones, Joseph D. Roy-Mayhew

**Affiliations:** Markforged, Inc., Watertown, MA 02472, USA; jessica.faust@markforged.com (J.L.F.); peter.kelly@markforged.com (P.G.K.); bruce.jones@markforged.com (B.D.J.)

**Keywords:** fused filament fabrication, 3D printing, coefficient of thermal expansion, moisture, dimensional accuracy, carbon fiber reinforcement

## Abstract

Environmental effects—temperature and moisture—on 3D printed part dimensional accuracy are explored. The coefficient of thermal expansion of four different nylon materials was determined for XY and ZX print orientations, with 0°, 45°/−45°, and 90° infill patterns. Unreinforced nylon exhibited a thermal expansion coefficient of the same order regardless of condition (from 11.4 to 17.5 × 10^−5^ 1/°C), while nylons reinforced with discontinuous carbon fiber were highly anisotropic, for instance exhibiting 2.2 × 10^−5^ 1/°C in the flow direction (0° infill angle) and 24.8 × 10^−5^ 1/°C in the ZX orientation. The temperature profile of a part during printing is shown, demonstrating a build steady state temperature of ~ 35 °C. The effect of moisture uptake by the part was also explored, with dimensional changes of ~0.5–1.5% seen depending on feature, with height expanding the most. The effects of moisture were significantly reduced for large flat parts with the inclusion of continuous fiber reinforcement throughout the part.

## 1. Introduction

The 3D printing industry grew over 25% per year for the past decade, representing over $12B USD in 2020 in revenues [1]. Although additive manufacturing adoption initially centered around prototypes, Wohler’s recent survey reports that a plurality of respondents (>30%) now use additive manufacturing for end-use parts [1]. Fused filament fabrication (FFF) technology, which involves laying down a bead of thermoplastic in a layer-by-layer fashion, was pioneered over 30 years ago and has become a dominant 3D printing technology [2]. FFF, especially when reinforced with continuous fiber, has become a mainstay of tooling and fixture applications, and is finding its way into more and more end-use applications. As plastic part properties are highly dependent on the processing method used, for instance due to cooling rates or flow conditions, one may assume that bulk material properties from injection molded samples may not align with those of additively manufactured parts. Therefore, a strong understanding of the FFF process, parameters, and resulting microstructure is required to give confidence in the material properties and facilitate widespread adoption of end-use 3D printed parts.

Research into the mechanical performance of end-use parts based on a variety of FFF print parameters has been previously studied [3,4]. Ding et al. found higher nozzle temperatures increased tensile and flexural strength of 3D printed PEEK samples, a high-temperature semi-crystalline thermoplastic [5]. Attolico et al. studied the tensile strength of ABS and PLA dog bones with infill orientations of 0°, 45/−45°, and 90°. It was found that the 0° infill samples show the highest tensile strength for both materials, while 90° resulted in the lowest strength [6]. Similar results were found by Rakpurohit et al. for PLA parts with 0° infill angle [7]. Cwikla et al. studied various infill patterns and found the hexagonal infill yielded the highest tensile strength, whereas Akhoundi et al. found the concentric pattern had the highest strength due to a higher percentage of paths parallel with the loading direction [8,9]. Terekhina et al. found tensile strength to be proportional to infill density for nylon filaments with 45/−45° infill angle [10]. Ma, Faust, and Roy-Mayhew recently added to this discourse, showing how moisture and print orientation dominate mechanical properties in Markforged’s polymer system [11]. These studies focused on the function of an end-use part. Additionally important is the form and fit of parts, which are less explored in the academic literature although critically important.

Dimensional accuracy can be thought of as how close the 3D printed part matches the original CAD design, and part tolerance—in mm or percent—is a common specification for traditional manufacturing which can ensure part fit. Desired tolerances can be defined by international tolerance grades, such as IT7 and IT10 for traditional end-use processes. For parts with dimensions up to 100 mm, for example, IT7 (i.e., turning process) tolerances are ±0.035 mm, while IT10 (i.e., milling process) tolerances are ±0.14 mm [12]. For similar sized parts, fused filament fabrication (FFF) 3D printing can reach tolerances of ±0.5 mm for desktop printers (typically those priced < $1000 and used by hobbyists) and ±0.15 mm for industrial printers (typically priced > $10,000 and used in manufacturing environments) [13,14]. In FFF, dimensional accuracy is a function of the machine accuracy and repeatability, as well as intrinsic material properties of the thermoplastic used to form the part. To achieve high tolerances, FFF 3D printers need to be able to accurately and precisely position the nozzle to lay down material where expected and reliably extrude the expected amount of material. Although many industrial-grade printers claim good accuracy and tight tolerances during the printing process, a more detailed exploration is needed to ensure dimensional accuracy extends throughout the life and use of the part.

The thermal behavior of the material during and immediately after the printing process must be considered. Similar to those used in injection molding, thermoplastics typically used in FFF printing experience shrinkage as the plastic cools. However, in FFF, the part experiences a non-uniform temperature gradient due to its layer-by-layer fabrication. Thus, it is reasonable to expect that important factors driving part tolerances are (i) a material’s coefficient of thermal expansion (CTE), which in turn depends on the materials’ composition (filled or unfilled), (ii) printing factors such as print orientation, infill, the ratio between shells and infill, and (iii) the temperature of the end-use environment. Hygroscopic polymers also absorb moisture that can affect the dimensional accuracy of 3D printed parts, generally through swelling [15,16,17,18]. Like the CTE behavior of FFF materials, the moisture-driven dimensional accuracy is also dependent on the printing parameters. Therefore, understanding how temperature and moisture affect dimensional accuracy of the final part is key for the form, fit, function paradigm of end-use parts.

This paper studies the effects of CTE and moisture in the printed parts using both micro carbon fiber filled and unfilled nylon-based polymers to aid in the better design of accurate end-use parts. We detail the CTE of materials printed with a variety of different infill angles and print orientations, as well as describe the temperature profiles of parts during printing. We show the dimensional accuracy of parts conditioned in a dry and moist environment and compare these parts to the original CAD design for accuracy. Furthermore, we show how continuous carbon fiber reinforcement can stabilize part dimensions, especially in the case of large flat parts sensitive to warping from moisture absorption.

## 2. Materials and Methods

### 2.1. Materials and Printers

3D printing materials and printers used in this study were supplied by Markforged (Watertown, MA, USA). Three nylon-based filaments reinforced with micro carbon fibers (Onyx™, Onyx™ ESD, Onyx™ FR) and one nylon-based filament without carbon reinforcement (Nylon White) were tested. Selected parts were reinforced with continuous carbon fiber (Continuous Fiber—Carbon, CCF) as noted in the text. All parts were printed using Eiger™ software designed, developed, and provided by Markforged. The print bed was leveled using the print bed levelling utility. If a material change was conducted, the purge plastic nozzle utility was run three times to ensure the plastic nozzle was free from contaminants of the previous material. 

### 2.2. Coefficient of Thermal Expansion Studies

Rectangular tensile beams (10 mm width, 40 mm length, 1 mm thickness) of all four materials were printed on an X7^TM^ Industrial Printer for CTE measurements. Parts were printed in the XY orientation with 90°, 0°, or 45°/−45° infill and in the ZX orientation with 0° infill. The 90° and 45°/−45° infill orientations were not feasible to print due to the thin cross-section of the ZX beam. Samples were printed with and without shells to better understand the effects of shells on CTE behavior. One sample was printed for each orientation, infill, and shell condition. Post-printing, samples were transferred to a 9.5 L sealed dry box with four clay desiccant packs (2 Unit Pak, Desiccare, Inc., Las Vegas, NV, USA) to avoid absorption of moisture. All samples were tested in the dry state without further conditioning. 

Thermal expansion measurements were conducted on an Extar DMS6100 Dynamic Mechanical Analyzer (Seiko Instruments Inc., Chiba, Japan) using the tensile grip geometry. A static 100 mN tensile load was applied to the sample following ASTM E831 and heated from ambient temperature (~25 °C) to 150 °C. Heating rate was kept to 2 °C/min to ensure uniform temperature throughout the sample. Displacement vs. temperature data were plotted and the CTE value was calculated from the curve using the Muse Thermal Analysis software. Only the linear regions of the curve were used in calculations to avoid error due to thermal transitions in the base polymer. 

### 2.3. FLIR Camera and Test Setup

Temperature data were recorded during prints with a FLIR E96 thermal camera through a hole cut into the visor of a Markforged X7 Industrial Printer. For each test, a 50 mm × 50 mm × 50 mm cube was printed in Onyx at 200 μm layer heights and observed in 30 s intervals for the duration of the prints. The emissivity used for Onyx was 0.95.

### 2.4. Dimensional Accuracy Prints and Moisture Content

Dimensional accuracy was tested over a range of environmental conditions including steady state, dry, and 52% relative humidity (RH). Dimensional accuracy parts were printed using Onyx and Onyx ESD. Onyx ESD samples were printed with and without continuous carbon fiber (CCF) reinforcement. Steady state parts were exposed to ambient indoor atmosphere near Boston, Massachusetts for a minimum of six months to reach equilibrium conditions. Dry samples were placed in a vacuum oven at 75 °C for 44 h. Parts conditioned at 52% RH were placed in a 9.5 L sealed container with a saturated aqueous solution of magnesium nitrate hexahydrate (ACS grade, HiMedia Laboratories, Kennett Square, PA, USA) for 44 h. (aligning with ASTM D638 conditioning for mechanical testing). The temperature and humidity were measured with an EasyLog digital temperature and humidity sensor (EL-WiFi-TH+, Lascar Electronics, Erie, PA, USA). An additional sample was included through all of the conditioning cycles to measure moisture content at each step. Moisture measurements were collected with a Computrac Vapor Pro XL moisture analyzer (AMETEK Brookfield, Middleboro, MA, USA). In addition, the mass of each sample was recorded with a high-precision laboratory scale (AG204 DeltaRange, Mettler Toledo, Columbus, OH, USA) to verify moisture content throughout each conditioning state. 

### 2.5. Metrology

Dimensional accuracy and swelling behavior of Onyx™ ESD samples at steady state conditioning was investigated with FARO Arm (Model 21000) measurements. 3D point clouds were created using a Laser Line Probe HD camera and a 6 mm Zircon Ball Probe (MetrologyWorks, Blue Springs, MO, USA). Scans were analyzed with the PolyWorks Inspector Premium software (InnovMetric, Quebec, QC, Canada). Each sample was compared to the original CAD design and zeroed to the bottom face of the print to highlight any dimensional inaccuracies.

### 2.6. Microscopy

Part microstructure in the XY and ZX orientation was determined by taking optical microscopy of parts printed on a Mark Two printer. Samples were cross-sectioned and mounted in SamplKwik Fast Cure Acrylic potting media (Buehler, Lake Bluff, IL, USA) followed by polishing on an E-Prep 4 Grinding/Polishing System (Allied High Tech Products, Compton, CA, USA). The polishing process included a brief two-minute abrasion at 15 N and 600 RPM head/platen speed with each of 180, 320, 600, and 1200 grit sandpaper. The final polish was achieved with MasterMet Colloidal Silica Polishing Suspension (Buehler, Lake Bluff, IL, USA) for 9 min at 12 N. Samples were imaged with an upright materials microscope (DM2700 M, Leica Microsystems, Wetzlar, Germany) fitted with an HD digital camera (MC170 HD, Leica Microsystems, Wetzlar, Germany) at 50X and 100X magnification.

## 3. Results and Discussion

For end-use parts, geometric fit is important throughout the life of the part, in the environment the part is used. This environment includes both the temperature and the chemicals the part is exposed to. We first explore the coefficient of thermal expansion (CTE) and the printed structure–property relationship. We then examine the effects of CTE during printing by looking at the temperature profile of a part while it is printing. This is followed by the role of moisture in dimensional stability. This study concludes by looking at the mitigation of dimensional change with continuous fiber reinforcement through point cloud scans of end-use parts for electronics assembly lines.

### 3.1. Coefficient of Thermal Expansion

The layer-by-layer process of FFF 3D printing inherently creates an anisotropic microstructure. Therefore, it comes as no surprise when parts show anisotropic mechanical and thermal properties. In our previously reported work, in-depth studies on various print parameters show the build orientation is a key driver in the final mechanical performance of the material, and this aligns well with previously reported research on the mechanical properties of 3D printed parts [3,4,5,11,19]. However, there remains important areas in the design process that are less understood, such as the materials behavior and dimensional accuracy at elevated temperatures. CTE will influence both the dimensional accuracy during the printing process, when the material experiences a large decrease in temperature from the print head to the chamber temperature, as well as in end-use applications in heated environments. In addition, a wide variety of 3D printing materials exist including those with 2D filler to maximize the mechanical properties. The addition of 2D filler further complicates the thermal response of the material due to the filler’s ability to preferentially align along the print path. Therefore, understanding the CTE response in the X, Y, and Z directions for 3D printed materials, both with and without filler, is an important design parameter to achieve dimensionally accurate parts.

**Figure 1:**
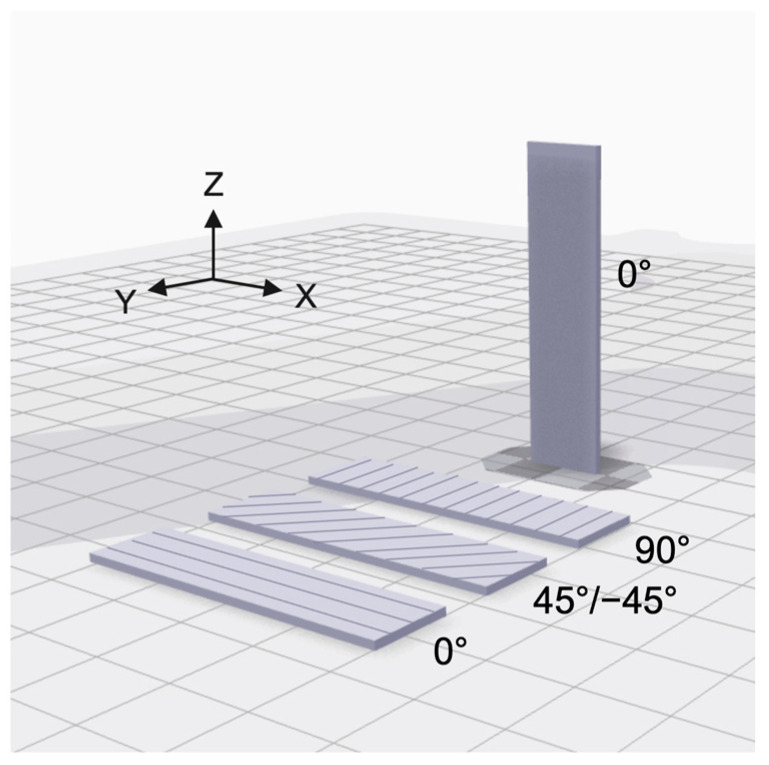
Schematic diagram showing print orientations with respect to the build plate and corresponding infill angles for each orientation.

CTE was measured to understand how both the base material and the print parameters affect the thermal expansion. Small rectangular parts of various filled and unfilled nylons (10 mm width, 40 mm length, 1 mm thickness) were printed on in the XY and ZX orientations, as shown in Figure 1. In the XY print orientation, parts were printed with 45°/−45° infill angle, the standard print setting in the Eiger Software. Parts were also printed with 0° and 90° infill to better understand the thermal expansion behavior in the flow (0°) and cross-flow (90°) directions of the printed bead. Testing the CTE in the flow and cross-flow directions helps to understand the effects of discontinuous carbon fiber on the overall CTE both in the direction of fiber alignment, or print path, and transverse to fiber alignment. In addition, understanding this behavior aids in CTE calculations of a final printed part with exterior shells, or continuous print paths around the exterior of the part.

As seen in Table 1, the CTE of Onyx is not highly dependent on temperature, averaging 3.6 × 10^−5^ 1/°C. Often polymers show two distinct slopes in CTE measurements; however, we did not see that phenomenon. Results show that the CTE is dependent on the print orientation of the part, as shown in Table 2. The Onyx-based materials with discontinuous carbon fiber reinforcement show a lower CTE in the XY orientation and a higher CTE in the ZX orientation. Parts printed in Nylon White without the micro carbon fiber reinforcement show more isotropic properties—albeit higher CTE—in all print orientations and infill patterns. These results align with previously reported CTE values for filled and unfilled FFF polymers where unfilled polymers generally show more isotropic CTE properties than their filled counterparts [20,21,22,23].

**Figure 2:**
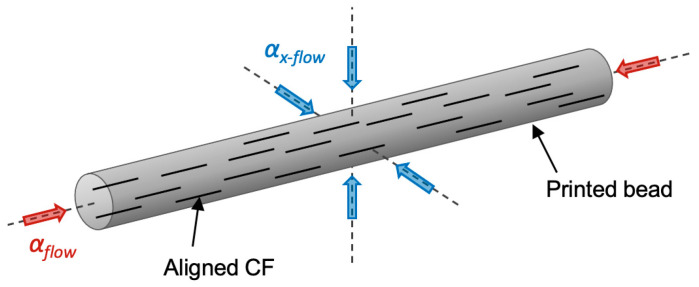
Schematic representation of a printed bead with aligned discontinuous carbon fiber showing the flow and cross-flow directions.

The CTE values for all micro carbon-reinforced filaments including Onyx, Onyx FR, and Onyx ESD were also found to be dependent on the infill angle within the part. This was most apparent when comparing the 0° infill angle to the 90° infill angle, representing the flow and cross-flow directions of the print path. In all reinforced materials, the CTE in the flow direction was lower than the cross-flow, consistent with the anisotropic properties of the carbon fiber reinforcement in the longitudinal and transverse direction. Previously reported literature states that the CTE of carbon fiber in the longitudinal direction is close to zero or negative, and positive in the transverse direction [24,25]. Alignment of carbon fiber within the printed bead during extrusion results in different CTE values in the final part based on carbon fiber orientation within the printed bead, as depicted in Figure 2. Therefore, the overall CTE of the final part will be dependent on the infill orientation of the internal layers. Parts can be printed with the 45°/−45° infill angle to create a more isotropic CTE and mechanical behavior in the XY plane due to the ability for each 45° aligned layer to counteract each −45° layer. In addition, Onyx FR and Onyx ESD showed more isotropic properties than Onyx due to the addition of flame-retardant materials and conductive filler, respectively.

Another factor that may contribute to enhanced expansion in the cross-flow direction is the potential for micro-porosity between adjacent printed beads, a structure often observed in FFF materials. Such porosity would provide a buffer zone into which material can freely expand, mitigating the overall expansion observed at the macro scale.

A larger value CTE was observed in the Z direction than any recorded in the XY plane. This may be due to the superposition of the two factors described above, the Z direction is transverse to the carbon fiber orientation, and there is no micro-porosity at the interface between vertically stacked beads to mitigate any of this expansion.

3D printed parts are often printed with shells to allow for sparse infill and to improve surface roughness. However, the path alignment of the shells varies from the infill and therefore introduces an additional variability in the CTE measurements. The effects of the shells on the thermal behavior of the overall part were tested by printing CTE samples with shells and varying infill angles. It is worth noting that the CTE samples used in this study are small (10 mm × 1 mm × 40 mm), and therefore the addition of the shells takes up a larger percentage of the total cross-sectional area (16%) than larger, more typical, parts. This will, however, make changes in the CTE behavior more prominent. Results show that the addition of shells may reduce the anisotropic behavior of the printed part and help to stabilize the CTE, as shown in Table 3. The addition of shells in the 0° infill sample does not change the CTE value significantly due to shells being printed in the same direction of the infill and therefore the carbon fibers are aligned in the same direction. However, samples printed with 90° infill show a reduction in the CTE with the addition of shells. 

**Figure 3:**
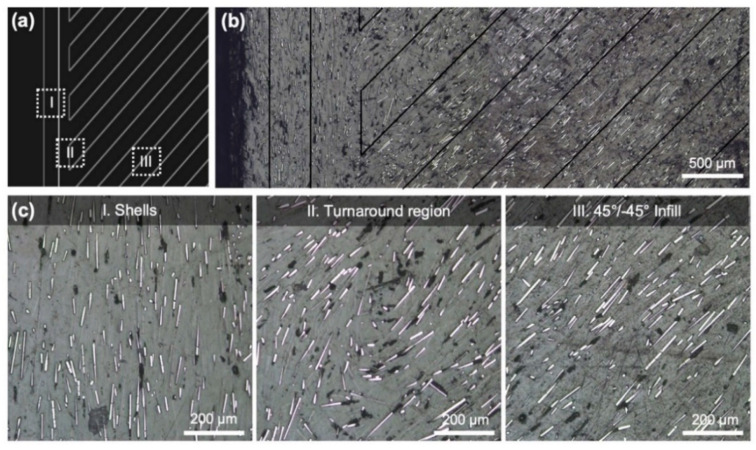
(**a**) Eiger 2D part showing variations of chopped carbon fiber alignment in the bulk (infill), turnaround, and shell region of the part. (**b**) Microscopy image of a polished cross-section with black lines added to indicate the tool paths. (**c**) Close-up microscopy image of fiber alignment in each corresponding region.

In the case of 45°/−45° infill, the CTE increased slightly. This was an unexpected result as a lower CTE was expected with the addition of the shells oriented at 0° to the CTE measurement direction. Therefore, the part was polished to reveal the internal structure and alignment of the discontinuous carbon fiber, as shown in Figure 3. It was found that in addition to the shell region that accounts for 16% of the total width (0.8 mm on each side), the path undergoes a turnaround region that takes up an additional 20% (1 mm on each side). Within this region, the discontinuous fibers are unaligned, and therefore it is difficult to predict the CTE in this region and the effects it has on the overall CTE. In the case of the 45°/−45° infill angle, the turnaround region may introduce a higher concentration of 90° aligned fibers and therefore increase the overall CTE. In the case of the 90° infill angle, the turnaround region will introduce additional 0° aligned fibers and therefore further reduce the overall CTE. This aligns with the measured results; however, more research is needed to extend this result for larger parts. In this study, the effects of the shells and turnaround regions are more prominent due to the small overall dimensions of the CTE sample. However, these effects are reduced as the part size increases and the CTE becomes predominantly based on the infill orientation. The overall feature size must be considered to successfully design parts for elevated temperatures.

### 3.2. Effects of CTE during Printing

**Figure 4:**
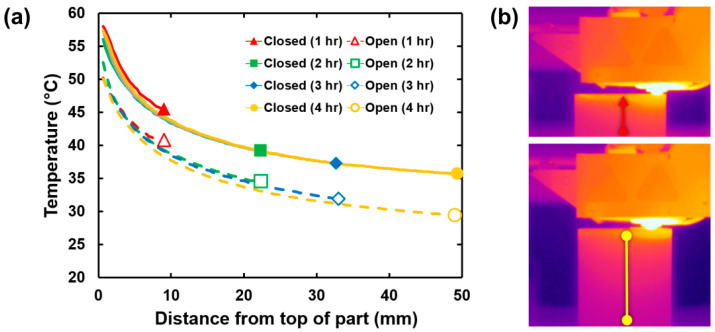
(**a**) Temperature profiles of test part surface with printer visor closed and open. (**b**) Parts are measured from top to bottom using FLIR Tools software. Red and yellow lines correspond to the points on (**a**).

In addition to understanding the CTE for the final end-use part, understanding how the CTE behaves during the printing process can aid in improving dimensional accuracy. We consider a few main phenomena. First, shrinkage of each path as it is printed and cools from printing temperature (generally > 200 °C) to the part temperature is expected. Second, a part will shrink as it cools from its steady state print temperature, or the chamber temperature, to ambient. Third, which was discussed above, is the dimensional change due to taking the part to a particular end-use environment. To better understand the first two phenomena, FLIR camera thermal data were used to plot the surface temperature profile of prints along their Z-axis. At the end of each hour of the print, the part temperature from top to bottom was measured and plotted e, as shown in Figure 4a,b. For example, at hour one (red solid triangle), the top of the part was ~58 °C, while the bottom of the part (10 mm from the top) was ~46 °C. At hour four (yellow solid circle), the top of the part was still ~58 °C, and 10 mm from the top was ~45 °C. The 40 and 50 mm from the top of the part sections—which physically correspond to the same plastic as measured in hour one, i.e., the bottom 10 mm of the part—both measure ~35 °C, showing that we are approaching a steady state temperature. This process was repeated with the printer chamber closed and open to test the sensitivity of the system and simulate the range of temperatures expected during the printing process.

For the closed door (manufacturer recommended) setup, each path can be expected to cool from the melt temperature to approximately 55 °C in the first few layers of the print, before slowly cooling and reaching the steady state chamber temperature of ~35 °C. However, it is important to note that smaller parts may never have segments that reach an equilibrium temperature while printing. By understanding these effects and coupling them with the specific material’s CTE, advanced slicers can compensate for this change and produce more dimensionally accurate parts. Nevertheless, changing the printing environment, for instance, by leaving the printer visor open, affects the part temperature profile. Although this is as expected as air can be readily exchanged with the ambient environment, manufacturers may not be accounting for this in their slicing software. For instance, Markforged print tuning and development are performed with the assumption that the visor is closed.

Part shrinkage, as it cools from steady state to ambient temperature, is readily calculated. For instance, the change in Z height caused by thermal contraction can be calculated using the above data and Equation (1), where *dL* is the change in length, *L*_0_ is the initial length, is the coefficient of thermal expansion of Onyx in the ZX orientation, *T*_1_ is the final temperature (ambient, 25 °C), and *T*_0_ is the initial temperature (steady state print temperature 35 °C).

(1) $$\;\;\;\;\;\;\;\;\;\;\;\;dL=L_{0}\alpha \left(T_{1}-T_{0}\right)$$

For the case of the 50 mm test cube printed in Onyx, in an idealized case, its theoretical ZX and XY shrinkage after printing are 124 μm (0.25%) and 18 μm (0.04%), assuming 45°/−45° infill with the visor closed. A similar calculation can be performed for additional end-use environments and print directions using the CTE data listed in Table 1, Table 2 and Table 3.

### 3.3. Role of Moisture in Dimensional Stability 

**Figure 5:**
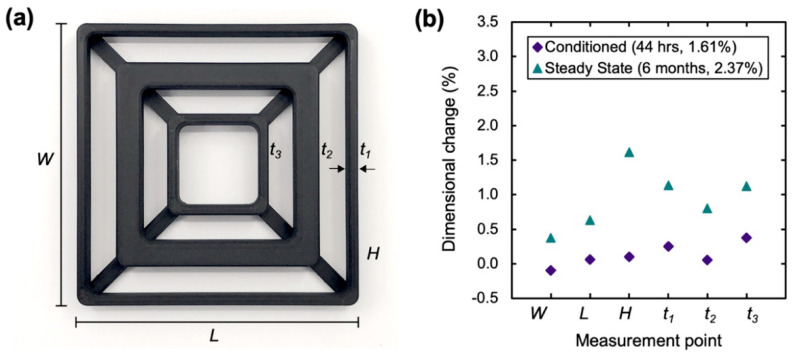
(**a**) Dimensional accuracy calibration part printed in Onyx with measurement points denoted. (**b**) Plot of moisture-induced swelling with respect to an Onyx dry state part. Values are the average of measurements taken from two independent samples and correspond to the labels in (**a**).

Similar to plastic parts made with traditional processes, 3D printed parts can deform over time in the environment, due to chemical, UV, or heat exposure. Temperature is clearly an important aspect of an end-use environment which affects part dimensions; however, a less considered factor is the humidity of the environment, which can cause part swelling. The effect of moisture on the thickness of six different features of a dimensional calibration part can be seen in Figure 5. In this study, the effects of temperature on dimensional accuracy during and after the printing process were negligible, as dimensional accuracy samples were conditioned in the steady state ambient environment for over 6 months prior to beginning dimensional accuracy testing. Interestingly, although it was shown previously that mechanical properties are rapidly affected by moisture uptake, showing an approximately 15% reduction in tensile strength after 44 h humidity conditioning, dimensional change requires greater exposure. For a 44 h conditioned sample, each measurement location swelled by less than 0.5%, with the change of many (*W*, *L*, *H*, *t*_2_) approaching the resolution of our micrometer (0.01 mm).

At a steady state condition (2.37% moisture weight gain), the critical features did not swell uniformly. The overall length and width swelled by approximately 0.5%, while beam thickness swelled by approximately 1%. The part height swelled the most, by approximately 1.5%. These changes are consistent with the level of reinforcement in the direction of expansion. Discontinuous fiber is aligned in the direction of W and L in all printed shells. For t1, t2, t3, infill patterns and 45°/−45° roof and floor printing provide partial alignment across the thickness of the part. However, there is no alignment with respect to the height dimension, where we see the greatest swelling. It is important to consider infill content and orientation, as similar externally looking struts may behave differently if their internal fill is different. Additionally, note that our observations are that the moisture-induced change is reversible, a part will revert to dry dimensions after a non-destructive drying procedure. The reversible behavior has potential applications in joining two parts with tightly toleranced dimensions based on moisture content and swelling behavior. 

### 3.4. Mitigation of Dimensional Change with Continuous Fiber Reinforcement

**Figure 6:**
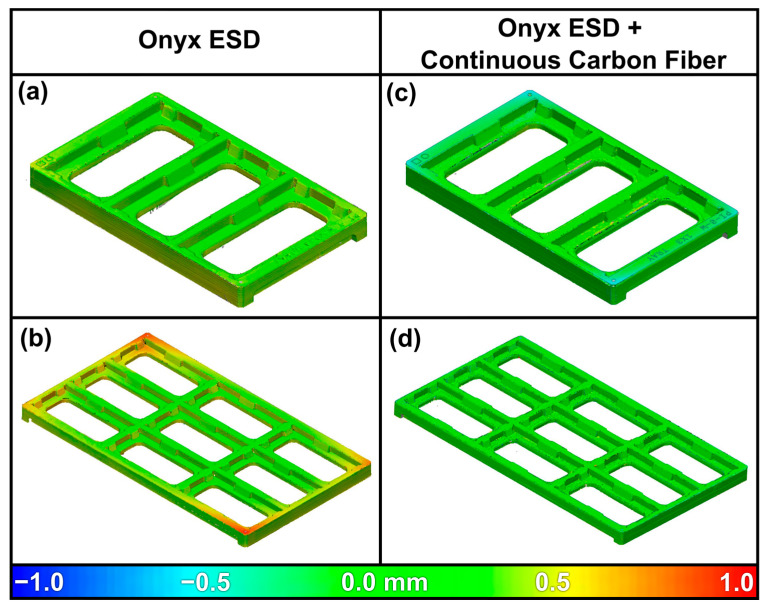
Onyx ESD part deviation from nominal (CAD specified) dimensions without (**a**,**b**), and with (**c**,**d**) continuous fiber reinforcement. The overall dimensions of these parts are 77 mm × 128 mm × 11 mm and 227 mm × 128 mm × 11 mm, respectively.

Although measuring individual dimensions can provide a snapshot of dimensional accuracy or part trueness, often a heat map is more useful to comprehend deviations across the part. Figure 6 shows such a heat map on a real part, showing deviations from CAD for PCB trays printed from Onyx ESD. In Figure 6a,b, one can see the strong adherence to the CAD in the center of the part, with increasing deviation towards the corners. Larger parts experience greater deviations, for instance, the nine cell tray experiences approximately 3× the deviation of the three cell tray—up to ~1 mm on a 227 mm × 128 mm × 11 mm part. In this case, internal stresses due to the printing process cause the part to curl in and warp up near the corners. Thin, large-area prints are most likely to experience this deformation. 

As can be seen in Figure 6c,d, reinforcing parts with continuous carbon fiber significantly improves the part dimensional accuracy, reducing stress-induced warping that is commonly seen in thin 3D printed parts [26,27]. Alternative techniques employed include printing brims on the parts, which although viable in some circumstances, increases the touch time per part to remove these features, and it reduces the printable area on the print bed. To improve dimension accuracy further, adaptive design, such as Blacksmith^TM^ software, can be used to account for and correct environmental and machine variances using user inputs, on-device sensors, and laser scanning systems.

## 4. Conclusions

End-use parts that retain shape in various environments can be made with continuous fiber-reinforced 3D printed parts. For plastic only, or discontinuous filler-reinforced plastic parts, the end-use environment—temperature and humidity—can contribute as much or more to final part tolerances than the part forming process. Absorbing over 2% moisture from the environment was shown to swell parts by 0.5–1.5% depending on the feature. This feature-dependent variation can be explained by alignment of the discontinuous carbon fiber within the printed bead, with the smallest swelling occurring along the direction of the print path and thus direction of reinforcement. The CTE and related thermal dimensional changes of Onyx, a discontinuous fiber filled nylon, were found to be highly dependent on print orientation—ranging from 2.2 × 10^−5^ 1/°C as aligned, to 9.5 × 10^−5^ 1/°C in cross-flow, and up to 24.8 × 10^−5^ 1/°C in the Z direction. This anisotropy was lower in materials with additional fillers, such as flame retardants in Onyx FR and conductive fillers in Onyx ESD, while an unreinforced material, Nylon White, showed the lowest anisotropy, but the largest average CTE ranging from 11.4 to 17.5 x10^−5^ 1/°C. Part dimensional accuracy can continue to be improved by incorporating the shrinkages expected during printing into the slicer. Additionally, adaptive intelligence that corrects for environmental and end-use variation can improve success of fit-to-function parts—on the first print. The exact relationship between microstructure and CTE anisotropy should be characterized in future work.

## Figures and Tables

**Table 1:** Detailed CTE measurements for Onyx with 45°/−45° infill from 25 to 150 °C.

Temperature Range [°C]	Coefficient of Thermal Expansion [10^−5^ 1/°C]
25–40	4.2
40–60	3.1
60–75	4.6
75–100	4.0
100–120	2.8
120–150	3.5
25–150 (overall)	3.6

**Table 2:** CTE values based on print orientation and infill angle.

Material	Coefficient of Thermal Expansion [10^−5^ 1/°C]			
	XY			ZX
	0° (Flow)	45°/−45°	90° (x-Flow)	0°
Onyx	2.2	3.6	9.5	24.8
Onyx FR	2.4	3.0	8.4	17.2
Onyx ESD	4.6	8.2	9.6	18.5
Nylon White	17.5	13.2	11.4	16.1

**Table 3:** Coefficient of thermal expansion values of Onyx with and without shells. All samples printed in the XY orientation.

Fill Angle	Coefficient of Thermal Expansion [10^−5^ 1/°C]	
	Onyx, Infill Only	Onyx, Infill + Shell
0°	2.3	2.2
45°/−45°	3.6	4.5
90°	9.5	6.6

## Data Availability

The data presented in this study are available on request from the corresponding author. The data are not publicly available due to involving proprietary materials.
